# Complete genome sequence of the filamentous anoxygenic phototrophic bacterium *Chloroflexus aurantiacus*

**DOI:** 10.1186/1471-2164-12-334

**Published:** 2011-06-29

**Authors:** Kuo-Hsiang Tang, Kerrie Barry, Olga Chertkov, Eileen Dalin, Cliff S Han, Loren J Hauser, Barbara M Honchak, Lauren E Karbach, Miriam L Land, Alla Lapidus, Frank W Larimer, Natalia Mikhailova, Samuel Pitluck, Beverly K Pierson, Robert E Blankenship

**Affiliations:** 1 Department of Biology and Department of Chemistry, Campus Box 1137, Washington University in St. Louis, St. Louis, MO 63130, USA; 2 Lawrence Berkeley National Laboratory & Production Genomics Facility, The DOE Joint Genome Institute, Walnut Creek, CA 94598, USA; 3 The DOE Joint Genome Institute and Bioscience Division, M888, Los Alamos National Laboratory, Los Alamos, NM 87544, USA; 4 Computational Biology and Bioinformatics Group, Biosciences Division, Oak Ridge National Laboratory, Oak Ridge, TN 37831, USA; 5 The DOE Joint Genome Institute, Walnut Creek, CA 94598, USA; 6 Department of Biology, CMB 1088, University of Puget Sound, Tacoma, WA 98416, USA; 7 Current: Baylor College of Medicine, Houston, TX 77030

## Abstract

**Background:**

*Chloroflexus aurantiacus *is a thermophilic filamentous anoxygenic phototrophic (FAP) bacterium, and can grow phototrophically under anaerobic conditions or chemotrophically under aerobic and dark conditions. According to 16S rRNA analysis, *Chloroflexi *species are the earliest branching bacteria capable of photosynthesis, and *Cfl. aurantiacus *has been long regarded as a key organism to resolve the obscurity of the origin and early evolution of photosynthesis. *Cfl. aurantiacus *contains a chimeric photosystem that comprises some characters of green sulfur bacteria and purple photosynthetic bacteria, and also has some unique electron transport proteins compared to other photosynthetic bacteria.

**Methods:**

The complete genomic sequence of *Cfl. aurantiacus *has been determined, analyzed and compared to the genomes of other photosynthetic bacteria.

**Results:**

Abundant genomic evidence suggests that there have been numerous gene adaptations/replacements in *Cfl. aurantiacus *to facilitate life under both anaerobic and aerobic conditions, including duplicate genes and gene clusters for the alternative complex III (ACIII), auracyanin and NADH:quinone oxidoreductase; and several aerobic/anaerobic enzyme pairs in central carbon metabolism and tetrapyrroles and nucleic acids biosynthesis. Overall, genomic information is consistent with a high tolerance for oxygen that has been reported in the growth of *Cfl. aurantiacus*. Genes for the chimeric photosystem, photosynthetic electron transport chain, the 3-hydroxypropionate autotrophic carbon fixation cycle, CO_2_-anaplerotic pathways, glyoxylate cycle, and sulfur reduction pathway are present. The central carbon metabolism and sulfur assimilation pathways in *Cfl. aurantiacus *are discussed. Some features of the *Cfl. aurantiacus *genome are compared with those of the *Roseiflexus castenholzii *genome. *Roseiflexus castenholzii *is a recently characterized FAP bacterium and phylogenetically closely related to *Cfl. aurantiacus*. According to previous reports and the genomic information, perspectives of *Cfl. aurantiacus *in the evolution of photosynthesis are also discussed.

**Conclusions:**

The genomic analyses presented in this report, along with previous physiological, ecological and biochemical studies, indicate that the anoxygenic phototroph *Cfl. aurantiacus *has many interesting and certain unique features in its metabolic pathways. The complete genome may also shed light on possible evolutionary connections of photosynthesis.

## Background

The thermophilic bacterium *Chloroflexus aurantiacus *was the first filamentous anoxygenic phototrophic (FAP) bacterium (also known as the green non-sulfur bacterium or green gliding bacterium) to be discovered [1]. The type strain *Cfl. aurantiacus *J-10-fl was found in a microbial mat together with cyanobacteria when isolated from a hot spring near Sokokura, Hakone district, Japan. *Cfl. aurantiacus *can grow phototrophically under anaerobic conditions or chemotrophically under aerobic and dark conditions.

The photosystem of *Cfl. aurantiacus *includes the peripheral antenna complex known as a chlorosome, the B808-866 light-harvesting core complex, and a quinone-type (or type-II) reaction center [2,3]. While *Cfl. aurantiacus *primarily consumes organic carbon sources (i.e. acetate, lactate, propionate, and butyrate) that are released by the associated cyanobacteria in the *Chloroflexus*/cyanobacterial mats of its natural habitat, it can also assimilate CO_2 _with the 3-hydroxypropionate (3HOP) autotrophic carbon fixation cycle [4,5]. Further, studies have reported carbon, nitrogen and sulfur metabolisms of *Cfl. aurantiacus *[1].

**Figure 1:**
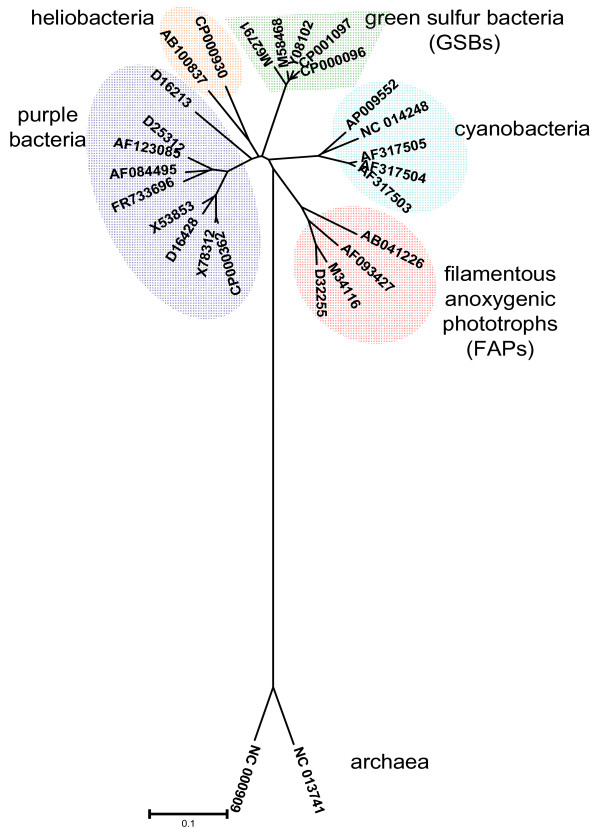
**Phylogenetic tree of photosynthetic bacteria**. The tree was constructed using the phylogenetic software MEGA4.1 with un-rooted neighbor joining 16S rRNA dendrogram from five phyla of photosynthetic microbes, including cyanobacteria, heliobacteria, purple bacteria, green sulfur bacteria and filamentous anoxygenic phototrophs (FAPs) (each phylum of bacteria highlighted in different color). Bacterial names and accession numbers of 16S rRNA genes are listed as follows: (1) purple bacteria: *Roseobacter denitrificans *OCh114 (CP000362), *Roseobacter litoralis *(X78312), *Rhodobacter capsulatus *(D16428), *Rhodobacter sphaeroides *2.4.1 (X53853), *Rhodopseudomonas faecalis *strain gc (AF123085), *Rhodopseudomonas palustris *(D25312), *Rhodopseudomonas acidophila *(FR733696), *Rhodopseudomonas viridis *DSM 133 (AF084495), *Rubrivivax gelatinosus *(D16213); (2) heliobacteria: *Heliobacterium gestii *(AB100837), *Heliobacterium modesticaldum *(CP000930); (3) cyanobacteria: *Oscillatoria amphigranulata *strain 19-2 (AF317504), *Oscillatoria amphigranulata *strain 11-3 (AF317503), *Oscillatoria amphigranulata *strain 23-3 (AF317505), *Microcystis aeruginosa *NIES-843 (AP009552), *Nostoc azollae *0708 (NC_014248); (4) green sulfur bacteria: *Chlorobaculum thiosulfatiphilum *DSM 249 (Y08102), *Pelodictyon luteolum *DSM 273 (CP000096), *Chlorobium limicola *DSM 245 (CP001097), *Chlorobaculum tepidum *TLS (M58468), *Chlorobium vibrioforme *DSM 260 (M62791); and (5) FAPs: *Chloroflexus aurantiacus *J-10-fl (M34116), *Chloroflexus aggregans *(D32255), *Oscillochloris trichoides *(AF093427), *Roseiflexus castenholzii *DSM 13941 (AB041226). Archaea (*Archaeoglobus profundus *DSM 5631 (NC_013741) and *Methanocaldococcus jannaschii *DSM 2661 (NC_000909)) were used as an out-group.

According to 16S rRNA analysis, *Chloroflexi *species are the earliest branching bacteria capable of photosynthesis [6-8] (Figure 1) and have long been considered to be critical to understanding the evolution of photosynthesis [9-16]. However, there are also indications that there has been widespread horizontal gene transfer of photosynthesis genes, so the evolutionary history of photosynthesis is still poorly understood [17].

During the transition from an anaerobic to an aerobic world, organisms needed to adapt to the aerobic environment and to become more oxygen-tolerant. Most of the gene products can function with or without oxygen, whereas several proteins and enzymes are known to be exclusively functional in either aerobic or anaerobic environments. Thus, gene replacements have been found in the evolution of many metabolic processes [18-20]. Some aspects of the genome annotation of *Chloroflexi *species have been discussed by Bryant, Ward and coworkers [5,21].

Several genes encoding aerobic and anaerobic enzyme pairs, as well as a number of duplicated gene clusters, have been identified in the *Cfl. aurantiacus *genome. In this report, we use genomic annotation, together with previous physiological and biochemical studies, to illustrate how *Cfl. aurantiacus *may be a good model system for understanding the evolution of metabolism during the transition from anaerobic to aerobic conditions. Some of the genomic information is compared with that of the genome of *Roseiflexus castenholzii*, a recently characterized FAP bacterium that lacks chlorosomes [22].

## Results and Discussion

### Genome properties

**Figure 2:**
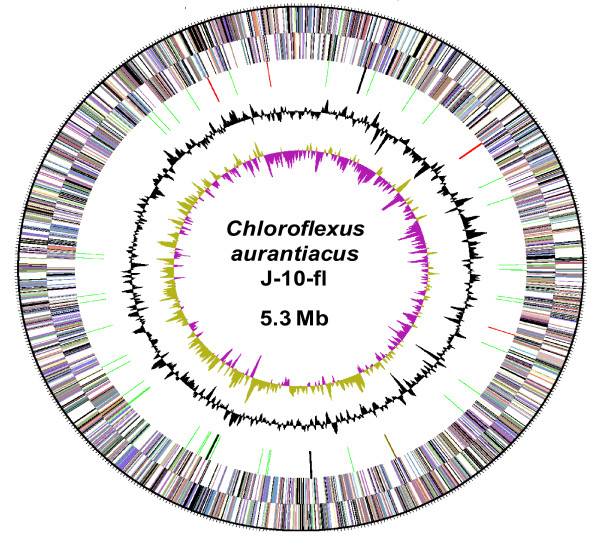
**Circular genome map of the 5.2-Mb *Cfl. aurantiacus *chromosome**. From outside to the center: Genes on forward strand (color by COG categories); Genes on reverse strand (color by COG categories); RNA genes (tRNAs, green; rRNAs, red; other RNAs, black); GC content; GC skew.

The genome size of *Cfl. aurantiacus *J-10-fl (5.3-Mb) (Table 1 and Figure 2) is comparable to that of other phototrophic *Chloroflexi *species: *Chloroflexus *sp. Y-400-fl (5.3-Mb), *Chloroflexus aggregans *(4.7-Mb), *Roseiflexus *sp. RS-1 (5.8-Mb), and *Roseiflexus castenholzii *DSM 13941 (5.7-Mb). Here, we summarize several unique features in the *Cfl. aurantiacus *genome, and compare some of the features with other *Chloroflexi *species and various photosynthetic and non-photosynthetic microorganisms. The complete genome has been deposited in GenBank with accession number CP000909 (RefSeq entry NC_010175). Further information is available at the Integrated Microbial Genome database (http://img.jgi.doe.gov/cgi-bin/pub/main.cgi?section=TaxonDetail&page=taxonDetail&taxon_oid=641228485). The *oriC *origin is at twelve o'clock of the circular genome map (Figure 2). Like in many prokaryotes, AT-rich repeated sequence can be found in the origin of replication.

**Table 1:** Organism information and genome statistics of *Chloroflexus aurantiacus *J-10-fl

Organism Information		
**Organism name**	***Chloroflexus aurantiacus *** **J-10-fl**	

External links	NCBI/RefSeq:NC_010175	

Lineage	Bacteria; Chloroflexi; Chloroflexi; Chloroflexales; Chloroflexaceae; Chloroflexus; aurantiacus	

Sequencing status	Finished	

Sequencing center	DOE Joint Genome Institute	

Oxygen requirement	Anaerobes	

Isolation	Hakone hot spring area in Japan	

Habitat	Fresh water, hot spring	

Motility	Mobile	

Temperature range (temperature optimum)	Thermophile (52-60°C)	

Cell shape	Filament-shaped	

Cell arrangement	filaments	

Gram staining	Gram-negative	

Phenotype	Green non-sulfur	

Energy source	Light (phototrophic growth) and organic carbon sources (chemotrophic growth)	

carbon assimilation	Photoautotrophy, photoheterotrophy, and chemoheterotrophy	

**Genome Statistics**		

	Number	Percentage of total genes or base pairs

DNA, total number of bases	5258541	100.00%

DNA G + C number of bases	2981443	56.70%

Total number of genes	3914	100.00%

Protein coding genes	3853	98.44%

- with function prediction	2845	72.69%

- without function prediction with similarity	1004	25.65%

- without function prediction without similarity	4	0.10%

Genes coding enzymes	934	23.86%

Genes coding fusion proteins	331	8.46%

Genes coding signal peptides	653	16.68%

Genes coding transmembrane proteins	789	20.16%

Pseudo genes	0	0.00%

RNA genes	61	1.56%

rRNA genes	9	0.23%

tRNA genes	49	1.25%

No special replication patterns can be found in the genome, and genes responsible for DNA replication and repair do not form a cluster (e.g., *dnaA *(Caur_0001), *dnaB *(Caur_0951), genes encoding DNA polymerase III (Caur_0523, Caur_0987, Caur_1016, Caur_1621, Caur_1930, Caur_2419, Caur_2639, Caur_2725 and Caur_3069), *polA *(Caur_0341), genes encoding other DNA polymerases (Caur_1575, Caur_1899, Caur_2099, Caur_2101, Caur_2868, Caur_3077, Caur_3225, Caur_3495, Caur_3509 and Caur_3510), *recD *(Caur_0824), *recF *(Caur_3876), *recG *(Caur_0261), *recQ *(Caur_2049), *rho *(Caur_0274), *gyrA *(Caur_1241), *gyrB *(Caur_3009), *uvrD *(Caur_0724) and others).

### A. Photosynthetic antenna and reaction center genes

*Cfl. aurantiacus *has chimeric photosynthetic components, which contain characteristics of green sulfur bacteria (e.g., the chlorosomes) and purple photosynthetic bacteria (e.g., the integral-membrane antenna core complex surrounding a type II (quinone-type) reaction center), As the first FAP bacterium to be discovered, the excitation energy transfer and electron transfer processes in *Cfl. aurantiacus *have been investigated extensively [3]. During phototrophic growth of *Cfl. aurantiacus*, the light energy is first absorbed by its peripheral light-harvesting antenna, the chlorosome, which is a self-assembled bacteriochlorophyll complex (the major bacteriochlorophyll in chlorosomes is bacteriochlorophyll *c *(BChl *c*)) and encapsulated by a lipid monolayer. Energy is then transferred to the B808-866 light-harvesting core antenna complex, which is a protein-pigment complex associated with two spectral types of bacteriochlorophyll *a *(BChl *a*) (B808 and B866), through the baseplate of chlorosomes. The baseplate is a CsmA chlorosome protein-bacteriochlorophyll *a *(BChl *a*)-carotenoid complex (i.e. a protein-pigment complex) [23]. Finally, the excitons are transferred to the reaction center (RC), in which photochemical events occur. While both purple photosynthetic proteobacteria and *Cfl. aurantiacus *have a type II RC [24], the *Cfl. aurantiacus *RC is simpler than the purple bacterial RC [2] and contains only the L- and M-subunits (PufL and PufM), and not the H-subunit [25,26].

All of the genes encoding the B808-866 core complex (α-subunit (Caur_2090) and β-subunit (Caur_2091)) and RC (*pufM *(Caur_1051) and *pufL *(Caur_1052)) are present in the *Cfl. aurantiacus *genome. The *pufL *and *pufM *genes are fused in the *Roseiflexus castenholzii *genome. The arrangement of genes for the core structural proteins of the photosynthetic complexes is significantly different from that found in purple bacteria, where the *puf *(photosynthetic unit fixed) operon invariably contains the LH complex genes, the RC genes encoding for the L and M subunits and the tetraheme cytochrome associated with the reaction center (if present) [27].

Although proteins are not required for BChl *c *self-assemblies in chlorosomes, various proteins have been identified to be associated with the lipid monolayer of the *Cfl. aurantiacus *chlorosomes [3]. In addition to the baseplate protein CsmA, the chlorosome proteins CsmM and CsmN have been characterized [3], and used to be considered as the only two proteins associated with the chlorosome mono-lipid layer. Other chlorosome proteins have also been reported, either through biochemical characterization (CsmP (unpublished results in Blankenship lab from in-solution trypsin digestion of the *Cfl. aurantiacus *chlorosomes) and AcsF [28]) or genomic analysis by analogy to green sulfur bacteria (CsmO, CsmP, CsmY) [29]. Among these proteins, AcsF, a protein responsible for chlorophyll biosynthesis under aerobic and semi-aerobic growth conditions, was unexpectedly identified from chlorosome fractions during anaerobic and photoheterotrophic growth of *Cfl. aurantiacus *[28]. There has been some discussions as to whether AcsF is obligated to be associated with the chlorosomes [21,30], and the role of AcsF under anaerobic growth condition remains to be addressed, because it is an oxygen-dependent enzyme in other systems [31,32]. Although more chlorosome proteins have been identified recently, it is clear that CsmA, CsmM and CsmN are the most abundant proteins of the *Cfl. aurantiacus *chlorosomes. Genes encoding the experimentally identified and proposed chlorosome proteins are *csmA *(Caur_0126), *csmM *(Caur_0139), *csmN *(Caur_0140), *csmP *(Caur_0142), *csmO *(Caur_1311), and *csmY *(Caur_0356).

### B. Electron transport complex genes

**Figure 3:**
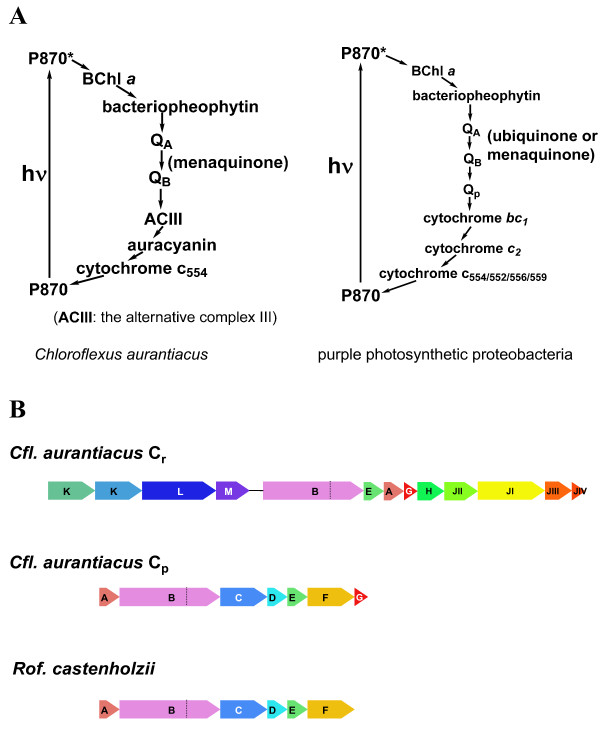
**Schematic representation of the proposed photosynthetic electron transport and the proposed ACIII operons**. The proposed photosynthetic electron transport in *Cfl. aurantiacus *(left) and in purple photosynthetic proteobacteria (right) (A), and the proposed ACIII operons in anaerobic photosynthesis (C_p_) and aerobic respiration (C_r_) in *Cfl. aurantiacus*, as well as the ACIII operon in *Roseiflexus *(*Rof*.) *castenholzii *(B). The characterized and putative proteins in the ACIII operon are listed as follows: **A**, multi-heme cytochrome *c*; **B**, MoCo Subunit (left) and FeS subunit (right); **C**, Integral membrane protein (polysulfide reductase, NrfD); **D**, uncharacterized protein; **E**, mono-heme cytochrome *c*; **F**, integral membrane protein; **G**, uncharacterized protein; **H**, electron transport SC01/SenC; **J**, cytochrome *c *oxidase subunits I-IV; **K**, FAD-linked oxidase; **L**, D-lactate dehydrogenase; and **M**, Cys and FeS rich domains. Abbreviation: BChl *a*, bacteriochlorophyll *a*; Q_A_, Q_B_, Q_p_, quinone-type molecules.

Figure 3A shows the proposed pathway of photosynthetic electron transport in *Cfl. aurantiacus *and purple photosynthetic proteobacteria. Similar to the purple photosynthetic proteobacteria, a cyclic electron transport pathway in *Cfl. aurantiacus *is also proposed. Nevertheless, some protein complexes in the electron transport chain of *Cfl. aurantiacus *are recognized to be substantially different from those of purple bacteria. *Cfl. aurantiacus *uses menaquinone as liposoluble electron and proton carrier [33-36], and purple proteobacteria use either ubiquinone [37,38] or menaquinone [39] as the mobile carrier in light-induced cyclic electron transport chain. The genetic information, analyses, and possible roles in photosynthesis and respiration for the complexes are described below.

#### (I) Alternative complex III (ACIII)

Integral membrane oxidoreductase complexes are essential for energy metabolism in all bacteria. In phototrophic bacteria, these almost invariably include the photoreaction center and a variant of respiratory Complex III, either the cytochrome *bc*_1 _complex (anoxygenic) or cytochrome *b*_6_*f *complex (oxygenic). No homolog of the Complex III has been identified biochemically in *Cfl. aurantiacus*, and no genes with significant homology to Complex III are found in the *Cfl. aurantiacus *genome. Previous experimental evidence indicated that alternative complex III (ACIII) complexes, identified in *Cfl. aurantiacus *and some non-phototrophic bacteria, function in electron transport [34,40-44]. Genes encoding an ACIII have also been identified in the genome of *Candidatus *Chloracidobacterium thermophilum [21], an aerobic phototrophic *Acidobacterium *[45]. In the *Cfl. aurantiacus *genome, two ACIII operons have been identified: one encodes the C_p _(subscript p stands for photosynthesis) ACIII complex for anaerobic photosynthesis, and the other encodes the C_r _(subscript r stands for respiration) ACIII complex for aerobic respiration (Table 2). The C_p _operon is similar to a seven-gene *nrf *operon in *E. coli *strain K-12. Hussain et al. suggested that the *nrf *operon in *E. coli *is essential for reducing nitrate to ammonia [46]. The *Cfl. aurantiacus *C_p _operon (Caur_0621 to Caur_0627) contains genes encoding two types of cytochrome *c*; a multi-heme cytochrome *c *(component A, *actA*, Caur_0621), which has recently been identified experimentally to be a penta-heme component [44], and a mono-heme cytochrome *c *(component E, *actE*, Caur_0625), which forms a homodimer in the ACIII complex [44], a putative FeS-cluster-hydrogenase component-like protein (component B, *actB*, Caur_0622), a polysulfide reductase (component C, *actC*, Caur_0623), similar to NrfD and likely involved in the transfer of electrons from the quinone pool to cytochrome *c*, an integral membrane protein (component F, *actF*, Caur_0626) and two uncharacterized proteins (component D (*actD*, Caur_0624) and component G (*actG*, Caur_0627)) (Figure 3B).

**Table 2:** Duplicate gene clusters of alternative complex III (ACIII) and NADH:quinone oxidoreductase (complex I) identified in the *Cfl. aurantiacus *genome

Gene products	Gene cluster 1	Gene cluster 2
Alternative complex III (ACIII)	*actABCDEFG *(Caur_0621 to Caur_0627) encoding C_p _(subscript p stands for photosynthesis) ACIII	Caur_2133 to Caur_2144 (12 genes) encoding C_r _(subscript r stands for respiration) ACIII

NADH:quinone oxido-reductase (complex I, EC 1.6.5.3)	*nuoA *to *nuoN *(Caur_2896 to Caur_2909)	*nuoA *(Caur_1987), *nuoB *(Caur_1986), *muoC *(Caur_1985), *nuoDE *(Caur_1984), *nuoF *(Caur_1185), *nuoH *(Caur_1982), *nuoI *(Caur_1983), *nuoJ *(Caur_1981), *nuoK *(Caur_1980), *nuoL *(Caur_1979), *nuoM *(Caur_1977 and Caur_1978) and *nuoN *(Caur_1976)

The proposed C_r _ACIII operon contains 12 genes (Caur_2133 to 2144) encoding a putative FAD-dependent oxidase (component K, *actK*, Caur_2133), D-lactate dehydrogenase (component L, *actL*, Caur_2134), a Cys-rich protein with Fe-S binding motifs (component M, *actM*, Caur_2135), components B (*actB*, Caur_2136), E (*actE*, Caur_2137), A (*actA*, Caur_2138), and G (*actG*, Caur_2139) in the C_p _ACIII operon, an electron transport protein SCO1/SenC (Caur_2140), and four subunits of cytochrome *c *oxidase (component J, Caur_2141 - 2144). The cytochrome *c *oxidase (COX, or complex IV, EC 1.9.3.1) genes in the C_r _operon are part of complex IV, so the C_r _ACIII operon clustered with complex IV genes could create a respiratory superoperon (Figure 3B). Additionally, a gene cluster encoding a putative SCO1/SenC electron transport protein (Caur_2423) and two COX subunits (Caur_2425 (subunit II) and Caur_2426 (subunit I)) is 300 genes away from the putative C_r _ACIII operon. Note that genes encoding components C (*actC*), D (*actD*) and F (*actF*) in the C_p _ACIII operon are absent in the C_r _ACIII operon. Whether these three components are required for the formation of the ACIII complex under aerobic respiratory growth will be addressed with biochemical studies.

#### (II) Auracyanin

Two type I blue copper proteins have been isolated and proposed to function as the mobile electron carriers in photosynthetic electron transport of photosynthetic organisms: one is plastocyanin in cyanobacteria, photosynthetic algae and higher plants and the other is auracyanin in *Chloroflexus *and *Roseiflexus*. The type I blue copper protein auracyanin, which has a single copper atom coordinated by two histidine, one cysteine and one methionine residues at the active site, is proposed to participate in the electron transfer from ACIII to the reaction center in *Cfl. aurantiacus *[35,47-49], and it has also been recently characterized in *Roseiflexus castenholzii *[50]. Additionally, an auracyanin gene (trd_0373) has been identified in the genome of the non-photosynthetic bacterium *Thermomicrobium roseum *DSM 5159, which is evolutionally related to *Cfl. aurantiacus *[51]. Two ACIII operons are proposed in *Cfl. aurantiacus*, and the two auracyanin proteins of *Cfl. aurantiacus*, AuraA and AuraB, which share 38% sequence identity, have been suggested to function with the two variant ACIII complexes [35]. AuraA, a water-soluble protein, can only be detected during phototrophic growth, whereas AuraB, a membrane-tethered protein, is synthesized during both phototrophic and dark growth [35]. It has been hypothesized that AuraA transports electron from the C_p _ACIII during photosynthesis and AuraB from the C_r _ACIII during respiration. The *auraA *(Caur_3248) and *auraB *(Caur_1950) genes are distant from the C_p _operon (Caur_0621 to Caur_0627) and C_r _operon (Caur_2132 to Caur_2144). In addition to *auraA *and *auraB*, two more genes encoding auracyanin-like proteins (or type I blue-copper proteins) (Caur_2212 and Caur_2581) have also been found in the *Cfl. aurantiacus *genome. In contrast, *Roseiflexus castenholzii *has only one copy of the ACIII operon (a six-gene cluster, Rcas_1462 to Rcas_1467), in which the gene encoding the component G of the *Cfl. aurantiacus *C_p _ACIII complex is missing) (Figure 3B), and one *auraA*-like gene (Rcas_3112).

#### (III) NADH:quinone oxidoreductase

Two operons encoding the enzymes for NADH:quinone oxidoreductase (Complex I, EC 1.6.5.3) are present in the genome. Complex I catalyzes electron transport in the oxidative phosphorylation pathway. Many bacteria have 14 genes (*nuoA *to *nuoN*) encoding Complex I, and some photosynthetic bacteria, such as the purple photosynthetic proteobacteria *Rhodobacter sphaeroides *and *Rhodopseudomonas palustris*, contain two Complex I gene clusters. In *Cfl. aurantiacus*, two putative Complex I gene clusters have been identified, one with all of the 14 gene subunits arranging in order (*nuoA *to *nuoN*, Caur_2896 - 2909), and the other has genes loosely arranged (with *nuoE *and *nuoF *800 genes apart), duplicated *nuoM *genes, and the lack of *nuoG *(Table 2). It is possible that either *nuoG *is shared with the two putative Complex I gene clusters or an alternative gene with less sequence similarity functions as *nuoG*. For example, two gene loci (Caur_0184 and Caur_2214) encoding gene products that have ~24% sequence identity with NuoG, which is a molybdopterin oxidoreductase. To date, there have been no biochemical studies on the Complex I from *Cfl. aurantiacus *or any FAP bacteria.

#### (IV) Other electron transport proteins

**Figure 4:**
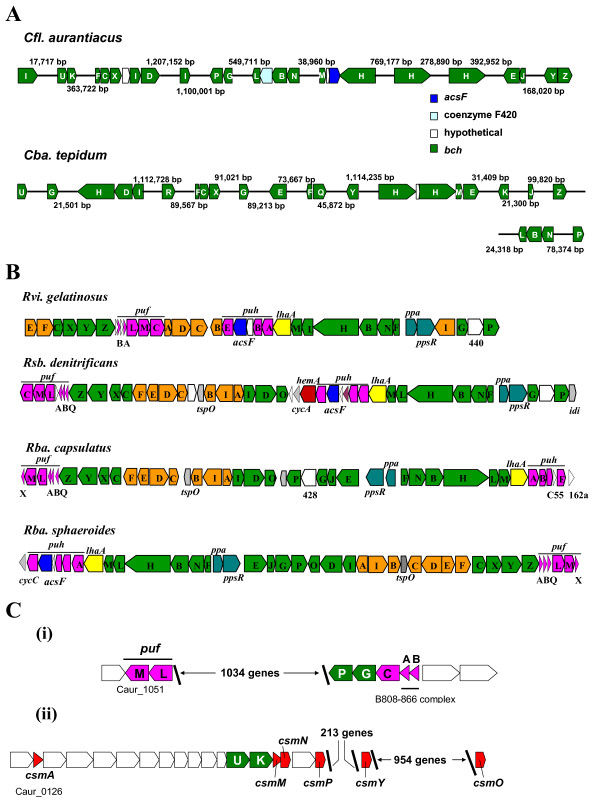
**Schematic representation of photosynthetic genes in photosynthetic bacteria**. Photosynthetic genes in *Cfl. aurantiacus*, *Cba. tepidum *(A), *Rba. sphaeroidies*, *Rba. capsulatus*, *Rvi. gelatinosus*, and *Rsb. denitrificans *(B), an operon consist of the *puf *genes (the L- and M-subunits of the reaction center and cytochrome c_554_), genes encoding two subunits of the B808-866 complex and two bacteriochlorophyll *a *biosynthesis enzymes (*bchP *and *bchG*) (i) and genes encoding bacteriochlorophyll *c *biosynthesis enzymes (*bchU *and *bchK*) and characterized and putative chlorosome proteins (*csmA*, *csmM*, *csmN*, *csmP*, *csmY *and *csmO*) (ii) (C). Genes are colored as listed: chlorosome proteins (*csm*) in red, chlorophyll biosynthesis (*bch*) in green, carotenoid biosynthesis (*crt*) in orange, reaction centers and light-harvesting complexes (*puf *and *puh*) in purple, regulatory proteins in blue (only in panel B), and uncharacterized and non-photosynthetic genes in white. All other gene colors are unique for clarity.

In addition to the electron transport proteins described above, the sequence has been determined of cytochrome *c*_554_, which is also a subunit of the reaction center of *Cfl. aurantiacus *[52-54]. The sequence of the cytochrome *c *subunit in the *Roseiflexus castenholzii *RC has also been reported [55]. The gene encoding cytochrome *c*_554 _(*pufC*, Caur_2089) is in an operon flanked with two genes encoding the bacteriochlorophyll biosynthesis enzymes, *bchP *(Caur_2087) and *bchG *(Caur_2088) at the 5'-end, and two genes encoding the B808-866 complex (Caur_2090 (α-subunit) and Caur_2091 (β-subunit)) at the 3'-end (Figure 4C).

### C. Aerobic/anaerobic enzyme pairs

#### (I) Tetrapyrroles

In the biosynthesis of heme and chlorophyll (Chl), three aerobic/anaerobic enzyme pairs participate: coproporphyrinogen III decarboxylase (aerobic, HemF (EC 1.3.3.3); anaerobic, HemN (EC 1.3.99.22)) and protoporphyrinogen IX oxidase (anaerobic and aerobic) in heme biosynthesis, and Mg-protoporphyrin IX monomethyl ester cyclase (aerobic, AcsF; anaerobic, BchE) in chlorophyll (Chl) biosynthesis. Both aerobic and anaerobic gene pairs can be found in *Cfl. aurantiacus*, for example, *acsF *(Caur_2590) and *bchE *(Caur_3676) [28] as well as *hemF *(Caur_2599) and *hemN *(Caur_0209 and/or Caur_0644) gene pairs. The *acsF *and *hemF *genes cannot be found in the green sulfur bacterium *Chlorobaculum tepidum *[56] and other strictly anaerobic bacteria. The genes involved in the biosynthesis of tetrapyrroles, as well as proposed gene replacements, are further elaborated below.

#### (a) Cobalamin

The gene replacements during the anaerobic to aerobic transitions are best known in the biosynthesis of cobalamin, in which the genes in the anaerobic pathway up to cobalt insertion into the corrin ring are completely replaced in the aerobic pathway [57]. Different strategies are used to generate cobyrinate diamide, the end product of both anaerobic and aerobic pathways, in which cobalt is introduced into the corrin ring at the dihydroisobacteriochlorin stage (early stage) of the anaerobic pathway and at the late stage of the aerobic pathway. The genomic information of *Cfl. aurantiacus *reveals a large cobalamin biosynthesis and cobalt transporter operon (Caur_2560 - 2580), containing genes in both aerobic and anaerobic biosynthesis pathways, suggesting that *Cfl. aurantiacus *can synthesize cobalamin under various growth conditions. Genes encoding anaerobic cobalt chelatase (EC 4.99.1.3) (*cbiK*, Caur_2572) and aerobic cobalt chelatase (EC 6.6.1.2) (*cobNST*, *cobN *(Caur_2579), *cobS *(Caur_1198) and *cobT *(Caur_2578)) have been identified (Tables 3 and 4). The aerobic cobalt chelatase (EC 6.6.1.2), containing three subunits (CobN, CobS and CobT), is a close analog to Mg-chelatase (also containing three subunits, BchH, BchI and BchD) that catalyzes the Mg-insertion in the chlorin ring in chlorophyll biosynthesis. It is known that aerobic cobalt chelatase subunits CobN and CobS are homologous to Mg-chelatase subunits BchH and BchI, respectively, and that CobT has also been found to be remotely related to the third subunit of Mg-chelatase, BchD. Compared to other strictly aerobic and anaerobic photosynthetic bacteria, the aerobic anoxygenic phototrophic proteobacterium *Roseobacter denitrificans *only carries the *cobNST *genes [27], and the strictly anaerobic bacterium *Heliobacterium modesticaldum *has only the *cbiK *gene [58]. The presence of *cobNST *and *cbiK *gene pairs in the *Cfl. aurantiacus *genome suggests a gene replacement in cobalamin biosynthesis by *Cfl. aurantiacus *under different growth conditions.

**Table 3:** Aerobic and anaerobic gene pairs identified in the *Cfl. aurantiacus *genome

(Putative) gene product and the EC number for aerobic and anaerobic gene product	Reaction catalyzed	Gene symbol and locus for aerobic genes	Gene symbol and locus for the anaerobic genes
**Tetrapyrrole (heme, cobalamin and chlorophyll) biosynthesis**			

coproporphyrinogen III decarboxylase/oxidase - aerobic (EC 1.3.3.3) - anaerobic (EC 1.3.99.22)	convert coproporphyrinogen III to protoporphyrinogen IX in heme biosynthesis	*hemF *(Caur_2599)	*hemN *(Caur_0209, Caur_0644)

Mg-protoporphyrin IX monomethyl ester oxidative cyclase (EC 1.14.13.81)	the isocyclic ring formation in chlorophyll biosynthesis	*acsF *(Caur_2590)	*bchE *(Caur_3676)

cobalt chelatase - aerobic (EC 6.6.1.2) - anaerobic (EC 4.99.1.3)	cobalt insertion on the corrin ring in cobalamin biosynthesis	*cobT *(Caur_2578), *cobN *(Caur_2579) *cobS *(Caur_1198)	*cbiK *(Caur_2572)

**Central carbon metabolism**			

α-ketoglutarate dehydrogenase E1 (EC 1.2.4.2), E2 (EC 2.3.1.61), and E3 (EC 1.8.1.4)	convert α-ketoglutarate to succinyl-CoA in the TCA cycle	E1 (*sucA*, Caur_3727) E2 (*olst*, Caur_1691, Caur_3726) E3 (*dld*, Caur_0170, Caur_2840)	

α-ketoglutarate:ferredoxin oxidoreductase (or α-ketoglutarate synthase) (EC 1.2.7.3)	convert succinyl-CoA to α-keto-glutarate in the TCA cycle		*korA *(Caur_0249, Caur_0952) *korB *(Caur_0250, Caur_0953)

pyruvate dehydrogenase E1 (EC 1.2.4.1), E2 (EC 2.3.1.12), and E3 (EC 1.8.1.4)	convert pyruvate to acetyl-CoA in pyruvate metabolism	E1 (Caur_1334, 1335, 1972, 1973, 2805, 3121, 3671, and 3672), E2 (Caur_1333, Caur_1974), E3 (*dld*, Caur_0170, Caur_2840)	

**Central carbon metabolism**			

pyruvate:ferredoxin oxidoreductase (or pyruvate synthase) (EC 1.2.7.1)	convert acetyl-CoA to pyruvate in pyruvate metabolism		*porA *(Caur_0249, Caur_0952, Caur_2080)

**Nucleic acid biosynthesis**			

ribonucleoside-diphosphate reductase aerobic (EC 1.17.4.1) anaerobic (EC 1.17.4.2)	convert ribonucleotide into deoxyribonucleotide in nucleic acids biosynthesis	*nrdB *(Caur_3331, β-subunit)	*nrdJ *(Caur_1750)

dihydroorotate oxidase (EC 1.3.3.1, aerobic), and dihydroorotate dehydrogenase (EC 1.3.99.11, anaerobic),	conversion of dihydroorotoate to orotate in pyrimidine biosynthesis	Caur_2081 and Caur_3923	Caur_2338

**Table 4:** Selected genes and gene clusters in metabolic pathways of *Cfl. aurantiacus*

Selected metabolic pathways and/or gene products	Gene symbol, gene loci, and/or gene products
CO oxidation	*coxSML *and *coxG *(Caur_3467 - 3470)

CO_2_-anaplerotic pathways	*tme *(Caur_1614), *pckA *(Caur_2331), *ppc *(Caur_3161, Caur_3888)

3-hydroxypropionate cycle	*accC *(Caur_1378, Caur_3421), *accA *(Caur_1647), *accD *(Caur_1648), *accB *(Caur_3739), *mcr *(Caur_2614), *pcs *(Caur_ 0613), *pccB *(Caur_2034, Caur_3435), *mcee *(Caur_3037), *mut *(Caur_1844, Caur_2508, Caur_2509), *smtA *(Caur_0179), *smtB *(Caur_0178), *sdhBAC *(Caur_1880 to Caur_1882), *fh *(Caur_1443), *mcl *(Caur_0174), *mch *(Caur_0173), *mct *(Caur_0174), *meh *(Caur_0180)

Glycolate assimilation and glyoxylate cycle	*glcDEF *(Caur_1144 - 1145, Caur_2133, Caur_2135), *glyr *(Caur_0825), *icl *(Caur_3889) and *mas *(Caur_2969)

Cobalamin biosynthesis	*cobS *(Caur_1198), *cobQ*/*cbiP *(Caur_2560), *N*-transferase (Caur_2561), *cobP*/*cobU *(Caur_2562), *cobB/cbiA *(Caur_2563), *cobA *(Caur_2564, Caur_0687), *cobJ *(Caur_2565), *cobM*/*cbiF *(Caur_2566), *cobI/cbiL *(Caur_2567), *cobL*/*cbiET *(Caur_2568), *cbiD *(Caur_2569), *cobH/cbiCH *(Caur_2570), *cbiK *(Caur_2572), *cobD*/*cbiB *(Caur_2573), *cbiMNQO *(Caur_2574 to Caur_2577), *cobU*/*cobT *(Caur_2578), *cobN *(Caur_2579), *cobO *(Caur_2580), *cysG *(Caur_0688) and *cbiMNQ *(Caur_3680 to Caur_3682) (Genes shared with aerobic and anaerobic pathways shown as aerobic/anaerobic)

Heme biosynthesis	*hemA*, *hemC*, *hemD*, *hemB*, *hemE*, *hemL*, *hemF *(Caur_2593 - 2599), *hemN *(Caur_0209 and Caur_0644), *hemG/Y *(Caur_0645), protoheme IX farnesyltransferase (Caur_0029)

Chlorophyll biosynthesis	*bchI *(Caur_0117, Caur_0419, Caur_1255), *bchU *(Caur_0137), *bchK *(Caur_0138), *bchF *(Caur_0415), *bchC *(Caur_0416), *bchX *(Caur_0417), *bchD *(Caur_0420), *bchP *(Caur_2087), *bchG *(Caur_2088), *bchBNL *(Caur_2554 to Caur_2557), *bchM *(Caur_2588), *acsF *(Caur_2590), *bchH *(Caur_2591, Caur_3151, Caur_3371)*, bchE *(Caur_3676), *bchJ *(Caur_3677), and *bchYZ *(Caur_3805, Caur_3806)

Nitrogen metabolism and amino acid biosynthesis	*hal *(Caur_0974), *tpl *(Caur_0573), *aspg *(Caur_3511), *glud*1 (Caur_1698, Caur_2070), *glul *(Caur_0844, Caur_1448, Caur_3395), *ilvA *(Caur_2585, Caur_3892), isoleucine/leucine/valine biosynthesis (Caur_0041, Caur_0163 - 0169, Caur_0329 - 0331, Caur_0488, Caur_1435, and Caur_2851)

Sulfur metabolism	Sulfur reduction operon (Caur_0686 - 0692), a sulfate adenylyl-transferase/adenylylsulfate kinase (Caur_2113), *cysK *(Caur_1341), *cysM *(Caur_3489), *sqr *(Caur_3894, type II SQR), sulfotransferase (Caur_2114).

B808-866 light-harvesting complex	Caur_2090 (α-subunit) and Caur_2091 (β-subunit)

Reaction center	*pufM *(Caur_1051) and *pufL *(Caur_1052)

Chlorosome proteins	*csmA *(Caur_0126)*, csmM *(Caur_0139)*, csmN *(Caur_0140), *csmP *(Caur_0142), *csmO *(Caur_1311) and *csmY *(Caur_0356).

Auracyanins (type I blue-copper proteins)	*auraA *(Caur_3248), *auraB *(Caur_1950), Caur_2212 and Caur_2581

Cytochrome c_554 _complex	*pufC *(Caur_2089)

Succinate dehydrogenase/fumarate reductase (complex II, EC 1.3.99.1)	*sdhBAC *(Caur_1880 - 1882)

Cytochrome *bc*_*1 *_or *b*_*6*_*/f *complex	Not annotated

Cytochrome *c *oxidase (complex IV, EC 1.9.3.1)	COX I - IV (Caur_2141 - 2144), COX I (Caur_2426) and COX II (Caur_2425 and Caur_2582)

#### (b) Heme

The heme operon (Caur_2593 to Caur_2599: *hemA*, hemC, *hemD*, *hemB*, *hemE*, *hemL *and *hemF*) is downstream of the cobalamin operon (Caur_2560 to Caur_2580). Except for *hemF*, other genes in the heme operon are utilized for synthesizing heme under both aerobic and anaerobic conditions. Furthermore, Caur_0209 and Caur_0644, encoding the putative O_2_-independent coproporphyrinogen III decarboxylase/oxidase (HemN, EC 1.3.99.22), and Caur_0645, encoding O_2_-dependent protoporphyrinogen oxidase (HemG/HemY, EC 1.3.3.4), are ~ 2000 genes away from the heme gene cluster. It is interesting to note that two *hemN *genes have been identified in the *Cfl. aurantiacus *genome, while no gene encoding the O_2_-independent protoporphyrinogen oxidase in *Cfl. aurantiacus *has been characterized. Because heme can be synthesized by *Cfl. aurantiacus *in both aerobic and anaerobic environments, it is possible that one of the *hemN *genes in *Cfl. aurantiacus *may encode an O_2_-independent protoporphyrinogen oxidase. Finally, in addition to protoheme (heme *b*), the genome predicts that heme *o *and heme *a *can be synthesized respectively by the gene products of Caur_0029 (encoding protoheme IX farnesyltransferase) and Caur_1010 (encoding a cytochrome *aa*3 biosynthesis protein), consistent with the spectral evidence provided by Pierson that protoheme and heme derivatives can be identified [59].

#### (c) (Bacterio)chlorophylls

The anaerobic to aerobic transitions are particularly intriguing in chlorophyll (Chl) biosynthesis and photosynthesis, in which molecular oxygen is lethal for photosynthetic anaerobes but is required for the life of aerobic phototrophs. Contrary to the cobalamin and heme biosynthesis, no gene cluster for (B)Chl biosynthesis is recognized in the *Cfl. aurantiacus *genome, whereas photosynthesis gene clusters are present in purple photosynthetic proteobacteria [27,60,61] (Figure 4B) and heliobacteria [58]. The photosynthetic genes of *Cfl. aurantiacus *are rather spread out in the chromosome, similar to the distribution of photosynthetic genes in *Cba. tepidum *(Figure 4A and Additional file 1: **Table S1**). Both aerobic and anaerobic genes, *acsF *(Caur_2590) and *bchE *(Caur_3676), have been identified in the *Cfl. aurantiacus *genome (Table 3), as well as in the genome of other phototrophic *Chloroflexi *species, including *Roseiflexus castenholzii *[28]. AcsF and BchE catalyze the isocyclic ring (or the E-ring) formation of Chl under aerobic and anaerobic growth conditions, respectively. AcsF (aerobic cyclase) catalyzes the formation of the isocyclic ring and reduces O_2 _into H_2_O. BchE requires cobalamin for catalytic activity, and has putative cobalamin and [4Fe-4S] cluster/*S*-adenosyl-*L*-methionine binding motifs [28,31,32,62-64].

Other anaerobic to aerobic transitions may also be found in the biosynthesis of BChls in *Cfl. aurantiacus*. For example, Mg-insertion to the porphyrin ring is the first committed step of BChl biosynthesis, and three *bchH *(Caur_2591, Caur_3151, and Caur_3371), three *bchI *(Caur_0117, Caur_0419 and Caur_1255) and one *bchD *(Caur_0420) have been annotated for the Mg-chelatase (BchHID) of *Cfl. aurantiacus*, whereas one each for *Hbt. modesticaldum *[58], *Rsb. denitrificans *[27] and several strictly anaerobic and aerobic bacteria. On the other hand, three *bchH*, and one copy of *bchD *and *bchI*, have been identified in the green sulfur bacterium *Cba. tepidum *(Additional file 1: **Table S1**), and Eisen et al. proposed [56] that different BchH gene products may contribute to synthesize different isoforms of BChl (BChl *a*, BChl *c*, and Chl *a *can be synthesized in *Cba. tepidum*). In comparison, two types of BChls, BChl *a *and BChl *c *can be synthesized by *Cfl. aurantiacus*. It is also possible that different *bchH *and *bchI *genes catalyze Mg-chelation to the BChl in various growth conditions of *Cfl. aurantiacus*.

Two *bchG*-like genes (*bchG *and *bchK*) encoding chlorophyll synthases that attach the tail into (bacterio)chlorophylls are present in the genome, as shown in Additional file 1: **Table S1**. Because the tails of BChl *a *(mostly phytyl- or geranylgeranyl-substituted) and BChl *c *(mainly stearyl-substituted) are rather distinct, it was suggested that one *bchG*-like gene encodes the enzyme synthesizing BChl *a *and the other homolog synthesizes BChl *c *[65]. The *bchG *gene sequence reported by Lopez et al. [65] was proposed to be BChl *a *synthase, since the encoding protein sequence is analogous to the sequence of chlorophyll synthase from *Rhodobacter capsulatus*. The proposed gene function was later verified [66]. The *bchK *gene encoding BChl *c *synthase was later confirmed with the *bchK*-knockout *Cba*. *tepidum *mutant [67]. Thus, *bchG *(Caur_2088) and *bchK *(Caur_0138) encode enzymes synthesizing BChl *a *and BChl *c*, respectively, in *Cfl. aurantiacus*. Although genes responsible for chlorophyll biosynthesis are rather spread out in the *Cfl. aurantiacus *genome, two genes responsible for BChl *c *biosynthesis, *bchU*, encoding C-20 methyltransferase [68], and *bchK*, are clustered with the genes encoding chlorosome proteins, and two BChl *a *biosynthesis genes, *bchP*, encoding geranylgeranyl hydrogenase [69], and *bchG*, are in the operon containing genes encoding cytochrome *c*_554 _(*pufC*) and the B808-866 light-harvesting complex (Figure 4C).

#### (II) Nucleic acids

The level of oxygen tolerance in *Cfl. aurantiacus *may be suggested from the presence of genes encoding ribonucleotide reductase (RNR), which is essential for DNA synthesis. Three classes of RNR have been reported, in which the class I is a diiron oxygen-dependent (NrdB, EC 1.17.4.1), class II is coenzyme B_12_-dependent (NrdJ, EC 1.17.4.2), and class III is *S*-adenosyl-*L*-methionine/[4Fe-4S] cluster-dependent (NrdG, EC 1.17.7.1). It has been suggested that biosynthesis of dNTP is catalyzed by NrdB, NrdJ, and NrdG in aerobic, aerobic and anaerobic, and strictly anaerobic environments, respectively. The activity of NrdJ in *Cfl. aurantiacus *has been reported [70]. Genes encoding NrdB and NrdJ, but not NrdG, have been found in the *Cfl. aurantiacus *genome, suggesting that NrdG and NrdJ produce dNTP for *Cfl. aurantiacus *in response to the oxygen level (Table 3). Moreover, in the fourth step of the biosynthesis of pyrimidine, the conversion of dihydroorotoate to orotate, dihydroorotate oxidase (EC 1.3.3.1, aerobic) versus dihydroorotate dehydrogenase (EC 1.3.99.11, anaerobic) are expressed in aerobic versus anaerobic conditions and genes encoding these enzymes have been identified (Table 3). Together, different classes of RNR and dihydroorotate oxidoreductase in the nucleic acid biosynthesis also suggest adaptation or evolution from anaerobic to aerobic conditions.

#### (III) Central carbon metabolism

Genes encoding several aerobic/anaerobic enzyme pairs in the central carbon metabolism, such as genes encoding pyruvate dehydrogenase (PDH, EC 1.2.4.1) and α-ketoglutarate dehydrogenase (KDH, EC 1.2.4.2), as well as pyruvate:ferredoxin oxidoreductase (or pyruvate synthase) (PFOR, EC 1.2.7.1) and α-ketoglutarate:ferredoxin oxidoreductase (or α-ketoglutarate synthase) (KFOR, EC 1.2.7.3) are present in the genome (Table 3). PFOR and KFOR, which are essential for pyruvate metabolism and energy metabolism through the TCA cycle, are commonly found in anaerobic organisms, whereas PDH and KDH are more widely spread and have been found in all aerobic organisms.

### D. CO_2 _assimilation and carbohydrate, nitrogen and sulfur metabolisms

#### (I) Carbon fixation and metabolism

**Figure 5:**
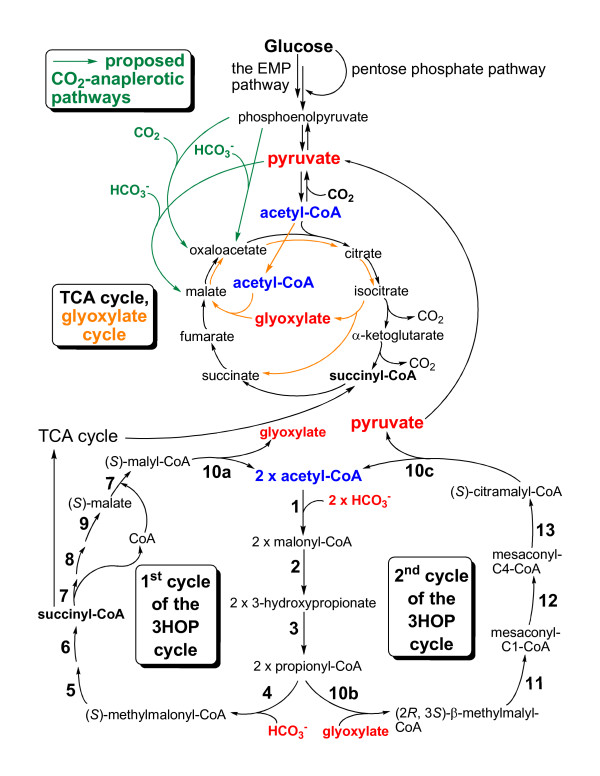
**Proposed autotrophic and anaplerotic CO**_**2 **_**assimilation and central carbon metabolic pathways of *Cfl. aurantiacus***. The enzymes required for each reaction step of the 3-hydroxypropionate (3HOP) autotrophic CO_2_-fixation cycle were described in the Results and Discussions.

Genes encoding carbon monoxide dehydrogenase (*coxG *and *coxSML*) have been found in the genome, suggesting that *Cfl. aurantiacus *can use CO as an electron source during aerobic or semi-aerobic growth. A similar mechanism has been suggested in the aerobic anoxygenic phototrophic proteobacterium *Rsb. denitrificans *[27]. CO_2 _generated from CO oxidation can be assimilated by *Cfl. aurantiacus *via the autotrophic carbon fixation cycle and/or the CO_2_-anaplerotic pathways. Under autotrophic growth conditions *Cfl. aurantiacus *is known to use a unique carbon fixation pathway: the 3-hydroxypropionate (3HOP) autotrophic cycle [4,5,71-74]. Three inorganic carbon molecules are assimilated into the 3HOP cycle to produce one molecule of pyruvate (Figure 5). A similar carbon fixation pathway called 3-hydroxypropionate/4-hydroxybutyrate (3HOP/4HOB) cycle was reported recently in archaea (Crenarchaeota) [75-78]. Several enzymes responsible for the 3HOP and 3HOP/4HOB cycles, including CO_2_-fixing enzymes (e.g., acetyl-CoA carboxylase and propionyl-CoA carboxylase), are common to the two pathways.

When the genome was first available, some genes required for the 3HOP cycle could not be found [5], whereas some of the missing genes/enzymes for the 3HOP cycle were later characterized experimentally [4]: (1) acetyl-CoA carboxylase (*accC *(Caur_1378 and Caur_3421, biotin carboxylase), *accA *(Caur_1647, α-subunit), *accD *(Caur_1648, β-subunit) and *accB *(Caur_3739) [79], (2) malonyl-CoA reductase (*mcr*, Caur_2614) [80], (3) propionyl-CoA synthase (*pcs*, Caur_0613) [81], (4) propionyl-CoA carboxylase (*pccB*, Caur_2034, Caur_3435), (5) methylmalonyl-CoA epimerase (*mcee*, Caur_3037), (6) *L*-methylmalonyl-CoA mutase (MCM) (*mut*, Caur_1844, Caur_2508, Caur_2509), (7) succinyl-CoA:(*S*)-malyl-CoA transferase (*smtA *(Caur_0179), *smtB *(Caur_0178)), (8) succinate dehydrogenase (*sdhBAC*, Caur_1880 to Caur_1882), (9) fumarate hydratase (*fh*, Caur_1443), (10) (*S*)-malyl-CoA/β-methylmalyl-CoA/(*S*)-citramalyl-CoA (MMC) lyase (*mcl*, Caur_0174), (11) β-methylmalyl-CoA dehydratase (*mch*, Caur_0173), (12) mesaconyl-C1-CoA-C4-CoA transferase (*mct*, Caur_0174) and (13) mesaconyl-C4-CoA hydratase (*meh*, Caur_0180) (Figure 5). The enzymes responsible for the 4^th ^step to the 6^th ^step of the 3HOP cycle are involved in fatty acid oxidation and amino acid metabolism. Finally, no genes encoding ribulose 1,5-bisphosphate carboxylase (RuBisCO) (the Calvin-Benson cycle), ATP citrate lyase (the reductive TCA cycle) and acetyl-CoA synthase (the Wood-Ljungdahl pathway) are present, strongly suggesting that these autotrophic carbon fixation pathways are not present in *Cfl. aurantiacus*.

Additionally, genes encoding malic enzyme (*tme*), phosphoenolpyruvate (PEP) carboxykinase (*pckA*) and PEP carboxylase (*ppc*) have been identified, suggesting that *Cfl. aurantiacus *can assimilate some CO_2 _and replenish the metabolites in the TCA cycle through the CO_2_-anaplerotic pathways. The active CO_2_-anaplerotic pathways have been identified experimentally in other anoxygenic phototrophs during autotrophic, mixotrophic and heterotrophic growth [82-87], and the activities of PEP carboxylase and malic enzyme have also been detected in cell extracts during photoheterotrophic growth of *Cfl. aurantiacus *(Tang and Blankenship, unpublished results). Moreover, all of the genes in the TCA cycle are present in the *Cfl. aurantiacus *genome.

In central carbon metabolism, all of the genes in the TCA cycle as well as the glyoxylate cycle have been identified. The glyoxylate cycle is one of the anaplerotic pathways for assimilating acetyl-CoA, thus lipids can be converted to carbohydrates. Glyoxylate is synthesized and also assimilated in the 3HOP cycle (Figure 5), and is also produced by isocitrate lyase (EC 4.1.3.1) (*icl*, Caur_3889) and consumed by malate synthase (EC 2.3.3.9) (*mas*, Caur_2969) in the glyoxylate cycle. With acetate as the sole organic carbon source to support the photoheterotrophic growth of *Cfl. aurantiacus*, higher activities of isocitrate lyase and malate synthase have been reported [88]. Further, some FAP bacteria have been shown to assimilate glycolate from their habitat [89]. As glycolate can be converted to glyoxylate by glycolate oxidase (*glcDEF*, EC 1.1.3.15) and glyoxylate reductase (*glyr*, EC 1.1.1.26), the glyoxylate shunt and the 3HOP cycle can be employed by *Cfl. aurantiacus *for assimilating glycolate. Together, genes encoding central carbon metabolism, 3HOP cycle, glycolate assimilation, the glyoxylate shunt and CO oxidation are listed in Table 4.

#### (II) Carbohydrate metabolism

Three carbohydrate metabolism pathways are utilized by various bacteria: the Embden-Meyerhof-Parnas (EMP) pathway (glycolysis), the Entner-Doudoroff (ED) pathway, and the pentose phosphate (PP) pathway. *Cfl. aurantiacus *does not have genes in the ED pathway, but has genes in the oxidative PP pathway, in agreement with the activities reported for the essential enzymes (glucose-6-phosphate dehydrogenase and 6-phosphogluconate dehydrogenase) in the oxidative PP pathway [90]. Genes in the non-oxidative pathway have also been found. The gene encoding fructose-1,6-bisphosphate (FBP) aldolase (EC 4.1.2.13), catalyzing the reaction of D-fructose-1,6-bisphosphate (FBP) ↔ glyceraldehyde-3-phosphate (GAP) + dihydroxyacetone phosphate (DHAP) in the EMP/gluconeogenic pathway, is missing in the genomes of *Chloroflexi *species (e.g., *Cfl. aurantiacus*, *Chloroflexus *sp. Y-400-fl and *Chloroflexus aggregans*). If *Cfl. aurantiacus *were unable to synthesize FBA, an active pentose phosphate pathway would be required for the interconversion of D-glucose-6-phosphate and GAP, so glucose and other sugars can be converted to pyruvate and other energy-rich species, and vice versa. However, *Cfl. aurantiacus *has been reported to grow well in glucose and a number of sugars during aerobic respiration [91], and uses the EMP pathway for carbohydrate catabolism [90]. Also, higher activities of phosphofructokinase and FBP aldolase have been found in the cells grown with glucose than with acetate [1,92,93]. Note that *Roseiflexi *species (e.g., *Roseiflexus *sp. RS-1 and *Roseiflexus castenholzii *DSM 13941), which are closely related to *Chloroflexi *species, have a putative bifunctional FBP aldolase/phosphatase gene identified [94], and genes encoding various types of aldolase have been found in the *Cfl. aurantiacus *genome. Taken together, *Cfl. aurantiacus *and other *Chloroflexi *species may employ either a novel FBP aldolase or more than one carbohydrate metabolic pathway. Further efforts will be needed to clarify this picture.

#### (III) Nitrogen metabolism and amino acid biosynthesis

*Cfl. aurantiacus *is known to use ammonia as the sole nitrogen source, and several amino acids (nitrogenous compounds), but not nitrate, can enhance the growth. Neither nitrogenase nor nitrogen fixation has been reported in *Cfl. aurantiacus *[95]. Consistent with the physiological studies, genes encoding enzymes in ammonia production, such as histidine ammonia lyase (*hal*), tyrosine phenol-lyase (*tpl*), asparaginase (*aspg*), glutamate dehydrogenase (*glud1*), and glutamate ammonia-lyase (*glul*), but not nitrogen metabolism (*nifDHK*) and nitrate reduction, are present in the genome. Note that *Cfl. aurantiacus *has genes encoding a copper-containing nitrite reductase (EC 1.7.2.1) (Caur_1570) and the α-subunit (*narG*, Caur_3201), but not other subunits (*narHIJ*) and the catalytic subunit (*nasA*), of nitrate reductase. Two threonine/serine dehydratases (EC 4.3.1.19), one of which is inhibited by isoleucine may be related to the isoleucine biosynthesis, and other key enzymes in isoleucine biosynthesis have been reported [96]. Consistent with the biochemical studies, two *ilvA *genes (Caur_2585 and Caur_3892) encoding threonine dehydratases, and genes in the isoleucine/leucine/valine biosynthesis pathway have been identified (Table 4). The biosynthesis of isoleucine has recently been discovered through the citramalate pathway in several microbes [97,98], while no gene encoded citramalate synthase (CimA, EC 2.3.1.182), required for the citramalate pathway, has been found in the *Cfl. aurantiacus *genome.

#### (IV) Sulfur assimilation and sulfate reduction

**Figure 6:**
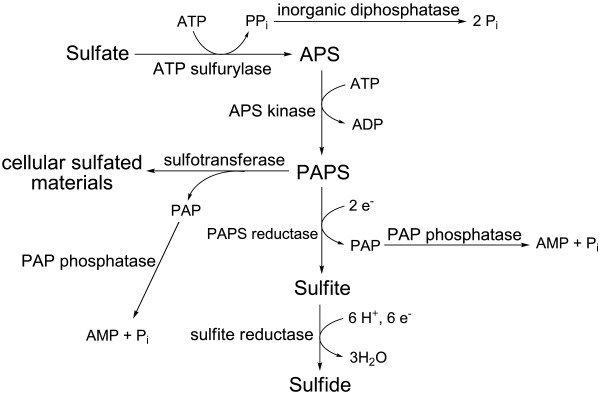
**Proposed sulfur reduction and assimilation pathways in *Cfl. aurantiacus***. All of the genes, except PAP phosphatase, have been identified in the genome.

*Cfl. aurantiacus *can use a variety of compounds as sulfur sources, including cysteine, glutathione, methionine, sulfide and sulfate, during photoheterotrophic or photoautotrophic growth [99,100]. When *Cfl. aurantiacus *uses sulfate as the sulfur source, high activity of ATP sulfurylase has been reported [99]. Sulfate is reduced to sulfide during photoautotrophic and photoheterotrophic growth for synthesizing cysteine and cofactors. Consistent with the experimental data, a complete sulfur reduction pathway with a sulfur reduction operon (Caur_0686 - 0692) has been identified (Table 4). Genes encoding two ATP sulfurylases (ATP + sulfate → adenosine 5'-phosphosulfate (APS) + PPi) can be identified: sulfate adenylyltransferase (EC 2.7.7.4, Caur_0690) and a bifunctional sulfate adenylyltransferase/adenylylsulfate kinase (Caur_2113). Pyrophosphate (PPi) produced in the reaction of ATP sulfurylase is hydrolyzed to inorganic phosphate (Pi) via inorganic diphosphatase (EC 3.6.1.1) (Caur_3321). The bifunctional enzyme or/and adenylylsulfate kinase (EC 2.7.1.25, Caur_0692) converts APS to 3'-phosphoadenosine 5'-phosphosulfate (PAPS), which is reduced to sulfite and PAP (adenosine 3',5'-diphosphate) by PAPS reductase (EC 1.8.4.8, *cysH*, Caur_0691). While many organisms reduce APS instead of PAPS to sulfite, it is unknown if *Cfl. aurantiacus *carries out this reaction as genes encoding APS reductase (EC 1.8.99.2) have not been identified in the genome. In addition to the proposed pathway, sulfotransferase (Caur_2114) can also transfer the sulfate group from PAPS, which serves as a sulfur donor, to an alcohol or amine acceptor for generating various cellular sulfate compounds. PAP is generated as a by-product in the reactions catalyzed by PAPS reductase and sulfotransferase, and has no known functions in metabolism and is likely hydrolyzed to AMP and Pi via PAP phosphatase (unidentified yet). Sulfite reductase (EC 1.8.1.2, Caur_0686) reduces sulfite to sulfide, which is incorporated into cysteine by cysteine synthase A (*cysK*, Caur_1341) or cysteine synthase B (*cysM*, Caur_3489). The overall proposed sulfur reduction and assimilation pathways are illustrated in Figure 6.

Other than using sulfide as the sulfur source during photoheterophic growth, *Cfl. aurantiacus *grows photoautotrophically in the presence of sufficient sulfide [100-102]. Under these circumstances, sulfide likely functions as electron donor by replacing organic carbon sources contributed from cyanobacteria. In agreement with these physiological and ecological studies, the gene encoding a type II sulfide:quinone oxidoreductase (SQR) (*sqr*, Caur_3894), has been found in the genome. SQRs belong to the members of the disulfide oxidoreductase flavoprotein superfamily. Other than type II SQRs, type I and type III SQRs with distinct sequences and structures and cofactor requirements have also been reported [103]. Although all of the characterized SQRs catalyze oxidization of sulfide to elemental sulfur (E(ox) + H_2_S → E_H2_(red) + S°), different types of SQR have been identified in various classes of photosynthetic bacteria [103]: type I, purple non-sulfur anoxygenic photosynthetic proteobacteria (such as *Rhodobacter capsulatus *) [104]; type II, *Cfl. aurantiacus *and cyanobacteria (such as *Synechocystis *PCC 6803) [103]; and type III, green sulfur bacteria [105]. In addition to being characterized in phototrophic microbes, type II SQRs have also been identified in various non-photosynthetic bacteria as well as in the mitochondria of animals, and are suggested to be involved in sulfide detoxification, heavy metal tolerance, sulfide signaling, and other essential cellular processes [103].

#### E. Evolution perspectives

**Figure 7:**
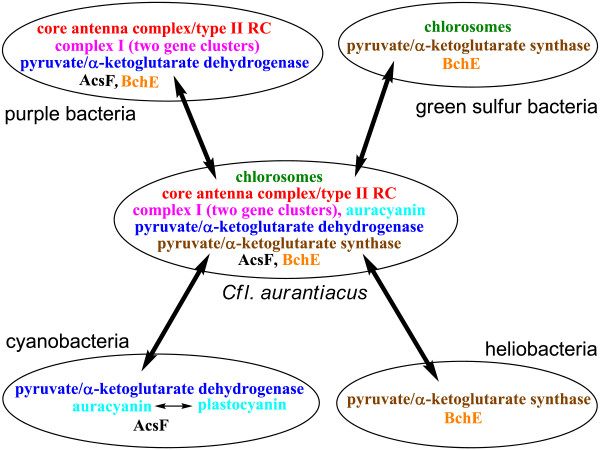
**Proposed lateral/horizontal gene transfers between *Cfl. aurantiacus *and other phototrophic bacteria**. Proposed gene transfers are shown in double-headed arrows. Genes in *Cfl. aurantiacus *may have been transferred either to or from other phototrophic bacteria. Genes encoding core antenna complex, type II reaction center (RC), pyruvate/α-ketoglutarate dehydrogenase, Complex I, AcsF and BchE may have been transferred from or to purple bacteria; pyruvate/α-ketoglutarate dehydrogenase, auracyanin and AcsF may have been transferred from or to cyanobacteria. Auracyanin may have been evolved from or to plastocyanin; chlorosomes, pyruvate/α-ketoglutarate synthase, BchE may have been transferred from or to green sulfur bacteria; and pyruvate/α-ketoglutarate synthase and BchE may have been transferred from or to heliobacteria.

Our paper reports numerous aerobic/anaerobic gene pairs and oxygenic/anoxygenic metabolic pathways in the *Cfl. aurantiacus *genome. As suggested by phylogenetic analyses [6-8] and comparisons to the genome and reports of other photosynthetic bacteria, one can propose lateral or horizontal gene transfers between *Cfl. aurantiacus *and other photosynthetic bacteria. Some proposed lateral gene transfers are listed below and also illustrated in Figure 7. Note that horizontal/lateral gene transfers suggested below are important in the evolution of photosynthesis. It is important to remember that it has not yet been generally accepted which organisms were donors and which were acceptors during gene transfers. The proposed gene transfers below remain to be verified with more sequenced genomes and biochemical studies in photosynthetic organisms.

#### (I) Photosynthetic components

Chlorosomes were transferred between *Cfl. aurantiacus *and the green sulfur bacteria (GSBs). The GSBs have larger chlorosomes and more genes encoding chlorosome proteins [21,29]. The integral membrane core antenna complex and a type II (quinone-type) reaction center were transferred either to or from the purple photosynthetic bacteria.

#### (II) (Bacterio)chlorophyll biosynthesis

AcsF and BchE are proposed to be responsible for biosynthesis of the isocyclic ring of (bacterio)chlorophylls under aerobic and anaerobic growth conditions, respectively [28,31,32,62-64]. The *acsF *gene was transferred either to or from purple bacteria and cyanobacteria, and the *bchE *gene either to or from heliobacteria, purple bacteria and GSBs.

#### (III) Electron transfer complexes

Two gene clusters of the complex I genes were transferred to (some) purple bacteria. Genes encoding auracyanin may have been transferred either to or from cyanobacteria where the type I blue copper protein plastocyanin is found. Alternative complex III (ACIII) may have evolved from or to the cytochrome *bc*_*1 *_or *b*_*6*_*/f *complex.

#### (IV) Central carbon metabolism

Genes encoding pyruvate dehydrogenase and α-keto-glutarate dehydrogenase were transferred either to or from purple bacteria and cyanobacteria, and genes encoding PFOR (or pyruvate synthase) and KFOR (or α-ketoglutarate synthase) to or from heliobacteria and GSBs. GSBs may have acquired the ATP citrate lyase gene to complete the reductive TCA (RTCA) cycle for CO_2 _fixation, and heliobacteria obtained the gene encoding (*Re*)-citrate synthase for synthesizing citrate and operating the partial oxidative TCA (OTCA) cycle [84]. Since *Cfl. aurantiacus *operates the OTCA cycle, the RTCA cycle in GSBs may have evolved from the OTCA cycle [106].

## Conclusions

The filamentous anoxygenic phototrophic (FAP) bacteria (or the green non-sulfur bacteria) have been suggested to be a critical group in the evolution of photosynthesis. As the first characterized FAP bacterium, the thermophilic bacterium *Chloroflexus aurantiacus *is an amazing organism. It has a chimerical photosystem that comprises characteristic types of green sulfur bacteria and purple photosynthetic bacteria. It is metabolically versatile, and can grow photoautotrophically and photoheterotrophically under anaerobic growth conditions, and chemotrophically under aerobic growth conditions. Consistent with these physiological and ecological studies, the *Cfl. aurantiacus *genome has duplicated genes and aerobic/anaerobic enzyme pairs in (photosynthetic) electron transport chain, central carbon metabolism, and biosynthesis of tetrapyrroles and nucleic acids. In particular, duplicate genes and gene clusters for two unique proteins and protein complexes in *Cfl. aurantiacus *and several FAP bacteria, the alternative complex III (ACIII) and type I blue copper protein auracyanin, have been identified in *Cfl. aurantiacus *genome. The genomic information and previous biochemical studies also suggest that *Cfl. aurantiacus *operates diverse carbon assimilation pathways. In contrast to the purple photosynthetic bacteria, the photosynthetic genes are rather spread out in the *Cfl. aurantiacus *chromosome. Overall, the genomic analyses presented in this report, along with previous physiological, ecological and biochemical studies, indicate that *Cfl. aurantiacus *has many interesting and certain unique features in its metabolic pathways.

## Methods

### Gene sequencing

The genome of *Chloroflexus aurantiacus *J-10-fl was sequenced at the Joint Genome Institute (JGI) using a combination of 8-kb and 14-kb DNA libraries. All general aspects of library construction and sequencing performed at the JGI can be found at http://www.jgi.doe.gov/. Draft assemblies were based on 58246 total reads. Both libraries provided 10x coverage of the genome. The Phred/Phrap/Consed software package (http://www.phrap.com) was used for sequence assembly and quality assessment [107-109]. After the shotgun stage, reads were assembled with parallel phrap (High Performance Software, LLC). Possible mis-assemblies were corrected with Dupfinisher [110] or transposon bombing of bridging clones (Epicentre Biotechnologies, Madison, WI). Gaps between contigs were closed by editing in Consed, custom primer walk or PCR amplification (Roche Applied Science, Indianapolis, IN). A total of 1893 additional reactions were necessary to close gaps and to raise the quality of the finished sequence. The completed *Cfl. aurantiacus *genome sequence contains 61248 reads, achieving an average of 11-fold sequence coverage per base with an error rate less than 1 in 100,000.

### Annotation

The genome of *Chloroflexus aurantiacus *J-10-fl has been annotated by the default JGI annotation pipeline. Genes were identified using two gene modeling programs, Glimmer [111] and Critica [112] as part of the Oak Ridge National Laboratory genome annotation pipeline. The two sets of gene calls were combined using Critica as the preferred start call for genes with the same stop codon. Briefly, structural RNAs were predicted using BLASTn and tRNAscan-SE [113] with default prokaryotic settings. Protein-coding genes were identified using gene modeling program Prodigal [114]. Genes with less than 80 amino acids which were predicted by only one of the gene callers and had no Blast hit in the KEGG database at 1e-05 were deleted. Predicted gene models were analyzed using GenePRIMP pipeline [115], and erroneous gene models were manually curated. The revised gene-protein set was searched by BLASTp against the KEGG GENES database [116] and GenBank NR using e-value of 1.0e-05, a minimum of 50% identity and alignment length of at least 80% of both the query and subject protein. These BLASTp hits were used to perform the initial automated functional assignments. In addition, protein sequences were searched against Pfam [117] and TIGRFAM [118] databases using HMMER2 package and trusted cutoffs for each model. Protein sequences were also searched against COG database [119] using RPS-BLAST search with e-value of 1.0e-05 and retaining the best hit. These data sources were combined to assert a product description for each predicted protein. Non-coding genes and miscellaneous features were predicted using tRNAscan-SE [113], TMHMM [120], and signalP [121]. The annotated genome sequence was submitted to GenBank and loaded into the Integrated Microbial Genomes (IMG) database [122].

### Phylogenetic analyses

The 16S rRNA gene sequences of various photosynthetic bacteria were obtained from NCBI. The sequences of 16S rRNA genes were aligned using the program Bioedit [123], and the phylogenetic tree was constructed using the program MEGA 4.1 [124]. The tree is an unrooted neighbor joining tree.

### Abbreviations of phototrophic bacteria

Three-letter abbreviation for the generic name of phototrophic bacteria follows the information listed on LPSN (List of Prokaryotic names with Standing in Nomenclature), an on-line database curated by professor Jean P. Euzéby (http://www.bacterio.cict.fr/index.html).

## Competing interests

The authors declare that they have no competing interests.

## Authors' contributions

BKP and REB designed research and provided DNA; KB, OC, ED, CSH, LJH, MLL, AL, FWL, NM and SP conducted sequencing, assembly and automated annotation; KHT, BMH, LEK and REB analyzed data; and KHT and REB wrote the paper. All authors read and approved the final manuscript.

## Supplementary Material

Additional file 1 **Table S1**. Annotation of photosynthetic genes in *Cfl. aurantiacus *and *Cba. tepidum*. Click here for file
