# Supplementary material for: Complete genome sequence of the filamentous anoxygenic phototrophic bacterium Chloroflexus aurantiacus
Source: BMC Genomics. 2011 Jun 29;12:334. doi: 10.1186/1471-2164-12-334 (PMC3150298; doi:10.1186/1471-2164-12-334)
Supplement: Additional file 1 — Table S1. Annotation of photosynthetic genes in Cfl. aurantiacus and Cba. tepidum. [file 1471-2164-12-334-S1.DOC]

**Supporting Information**

**Complete Genome Sequence of the Filamentous Anoxygenic Phototrophic Bacterium *Chloroflexus auranticus***

**Table S1. Annotation of photosynthetic genes in *Cfl. aurantiacus* and *Cba. tepidum***

| Gene  symbol | (Proposed) gene product | Gene loci in *Cfl. aurantiacus* | Gene loci in *Cba. tepidum* |
| --- | --- | --- | --- |
| *bchH* | magnesium chelatase, subunit H | Caur_2591, Caur_3151, and Caur_3371 | CT1295, CT1955, and CT1957 |
| *bchI* | magnesium chelatase, subunit I | Caur_0117, Caur_0419 and Caur_1255 | CT1297 |
| *bchD* | magnesium chelatase, subunit D | Caur_0420 | CT1296 |
| *bchB* | light-independent protochloro-phyllide reductase, subunit B | Caur_2556 | CT2151 |
| *bchN* | light-independent protochloro-phyllide reductase, subunit N | Caur_2557 | CT2152 |
| *bchL* | light-independent protochloro-phyllide reductase, subunit L | Caur_2554 | CT2150 |
| *bchX* | chlorophyllide reductase, subunit X | Caur_0417 | CT1423 |
| *bchY* | chlorophyllide reductase, subunit Y | Caur_3805 | CT1826 |
| *bchZ* | chlorophyllide reductase, subunit Z | Caur_3806 | CT2125 |
| *bchF* | 3-vinyl bacteriochlorophyllide hydratase | Caur_0415 | CT1421, CT1776 |
| *bchM* | Mg-protoporphyrin IX methyl transferase | Caur_2588 | CT1958 |
| *bchE* | magnesium-protoporphyrin IX methylester cyclase (anaerobic) | Caur_3676 | CT1697, CT1959 |
| *acsF* | magnesium-protoporphyrin IX methylester cyclase (aerobic) | Caur_2590 | N/A |
| *bchC* | 2-desacetyl-2-hydroxyethyl bacteriochlorophyllide *a* dehydrogenase | Caur_0416 | CT1422 |
| *bchU* | bacteriochlorophyll C-20 methyltransferase | Caur_0137 | CT0028 |
| *bchK* | bacteriochlorophyll *c* synthase | Caur_0138 | CT1610 |
| *chlG* | bacteriochlorophyll/chlorophyll *a* synthase | N/A | CT1270 |
| *bchG* | bacteriochlorophyll/chlorophyll *a* synthase | Caur_2088 | CT1992 |
| *bchP* | geranylgeranyl hydrogenase/ reductase | Caur_2087 | CT2256 |
| *bchQ* | bacteriochlorophyll C-8 methyl-transferase | N/A | CT1777 |
| *bchR* | bacteriochlorophyll C-12 methyltransferase | N/A | CT1320 |
| *bchJ* | 8-vinyl bacteriochlorophyllide reductase | Caur_3677 | CT2014 |

p.s. N/A: not annotated
